# High Prevalence of Human-Associated *Escherichia coli* in Wetlands Located in Eastern France

**DOI:** 10.3389/fmicb.2020.552566

**Published:** 2020-09-04

**Authors:** Daniel Martak, Charles P. Henriot, Marion Broussier, Charlotte Couchoud, Benoit Valot, Marion Richard, Julie Couchot, Gudrun Bornette, Didier Hocquet, Xavier Bertrand

**Affiliations:** ^1^Service d’Hygiène Hospitalière, Centre Hospitalier Universitaire, Besançon, France; ^2^UMR 6249, Laboratoire Chrono-Environnement, CNRS-Université de Bourgogne Franche-Comté, Besançon, France; ^3^Bioinformatique et big data au service de la santé, UFR Santé, Université de Bourgogne Franche-Comté, Besançon, France

**Keywords:** floodplains, wetlands, *E. coli*, human-associated *E. coli*, wastewater treatment plant

## Abstract

*Escherichia coli* that are present in the rivers are mostly brought by human and animal feces. Contamination occurs mostly through wastewater treatment plant (WWTP) outflows and field amendment with sewage sludge or manure. However, the survival of these isolates in river-associated wetlands remains unknown. Here, we assessed *E. coli* population structure in low-anthropized wetlands located along three floodplains to identify the major source of contamination of wetlands, whose functioning is different from the rivers. We retrieved 179 *E. coli* in water samples collected monthly from 19 sites located in eastern France over 1 year. Phylogroups B1 and B2 were dominant in the *E. coli* population, while phylogroup A was dominant in isolates resistant to third-generation cephalosporins, which harbored the extended-spectrum β-lactamase (ESBL) encoding genes *bla*_CTX–M–15_ and *bla*_CTX–M–27_ in half of the cases. The high proportion of isolates from human source can be attributed to WWTP outflows and the spread of sewage sludge. We analyzed the distribution of the isolates belonging to the most human-associated phylogroups (B2 and D) on a phylogenetic tree of the whole species and compared it with that of isolates retrieved from patients and from WWTP outflows. The distribution of the three *E. coli* populations was similar, suggesting the absence of a specific population in the environment. Our results suggest that a high proportion of *E. coli* isolates that reach and survive in low-anthropized environments such as wetlands are from human source. To the best of our knowledge, this is the first study assessing *E. coli* contamination and resistance genes in natural freshwater wetlands.

## Introduction

*Escherichia coli* is a facultative anaerobic organism commensal of the vertebrate gut microbiota. Paradoxically, it can also be responsible of infections and is one of the main pathogens responsible for hospital- and community-acquired infections (Tenaillon et al., 2010). Pathogenic strains of *E. coli* are divided into intestinal pathogens causing diarrhea and extraintestinal *E. coli* (ExPEC) causing a variety of infections in both humans and animals including urinary tract infections, meningitis, and septicemia (Johnson et al., 2003; Riley, 2014). Although the ExPEC major clone sequence type (ST) 131 became predominant and have spread worldwide, other clones are also frequent (i.e., ST10, ST38, ST69, ST73, and ST405) (Nicolas-Chanoine et al., 2014; Manges et al., 2019). In contrast, diarrheagenic *E. coli* have been clustered into different pathovars according to their virulence gene arsenal. Humans widely contribute to the dissemination of *E. coli* in the environment through wastewaters. In developed countries, these wastewaters are treated in wastewater treatment plants (WWTPs). However, WWTP outflows contain *E. coli* that are released to the rivers (Bréchet et al., 2014). Agriculture also contributes to *E. coli* dissemination through practices such as spreading manure or sewage sludge (Cabral, 2010; Niu and Phanikumar, 2015; Hocquet et al., 2016). Cattle also play an important role in the dissemination of *E. coli* in the environment through their feces (Cabral, 2010). Overall, *E. coli* contaminates nearly all environments, with both human-associated or not human-associated *E. coli* present in rivers, lakes, groundwater, plants, and soils (Brookes et al., 2004; John and Rose, 2005; Park et al., 2016).

*E. coli* clusters into phylogroups A, B1, B2, C, D, E, F, and G (Clermont et al., 2013, 2019). Strains belonging to phylogroups A and B1 are ubiquitous and adapted to humans or vertebrate animals. Phylogroup A strains are predominant in humans while B1 strains are predominant in animals (Berthe et al., 2013). The rare phylogroup C is closely related to phylogroup B1 (Moissenet et al., 2010). Strains belonging to phylogroups B2, D, and F are the most frequent phylogroups found in human ExPEC infections (Clermont et al., 2013).

Extended-spectrum β-lactamase (ESBL) is the major cause of resistance to cephalosporins in *E. coli*. In recent decades, ESBL-producing *E. coli* have spread worldwide, becoming a serious public health threat (Pitout and Laupland, 2008). These resistant strains have been widely isolated from humans but also from animals and food sources (Mughini-Gras et al., 2019; Pärnänen et al., 2019). In a One-Health perspective, many recent studies assessed the relationships between *E. coli* from humans, meat, livestock, and WWTPs to understand the role played by humans in the spread of *E. coli* (Day et al., 2019; Ludden et al., 2019). Here, we wanted to understand the fate of *E. coli* in low-anthropized environments by determining the *E. coli* population structure in three rivers and their associated wetlands. We further compared their population structure with those from clinical settings and of WWTP outflows to understand the dynamics of the *E. coli* phylogroups from the human source to the wetlands.

## Materials and Methods

### Bacterial Sampling

We sampled 16 wetlands distributed along the lower floodplains of the Ain, Doubs and Loue Rivers, three karstic rivers of the Jura Massif in eastern France (Supplementary Figure 1). One sampling point per river was chosen to compare the wetlands and the rivers. Each river and its associated wetlands were sampled monthly for 1 year (from March 2015 to March 2016). Samples of 250 mL of water were collected and 100 mL of water were filtered by 0.45 μm membranes and deposited on Drigalski agar plates (DRIGs) (Oxoid) and on ESBL-producing bacteria selection plates (chromID ESBL plates; bioMérieux, Marcy l’Etoile, France). After an overnight incubation at 37°C, colonies were counted; each lactose positive bacteria cultured on DRIGs and colonies growing on ESBL-specific plates were identified by MALDI-TOF MS (Microflex 100 LT; Bruker Daltonik GmbH, Bremen, Germany). All *E. coli* identified with a log value ≥ 2 according to the manufacturer’s recommendations and with different morphotypes were stored at −80°C until further analysis.

### Identification of *bla*_ESBL_ in Isolates Resistant to Third-Generation Cephalosporins (3GC-R)

For each morphotype of *E. coli* growing on chromID ESBL plates, we confirmed the production of ESBL with the synergy test recommended by the Antibiogram Committee of the French Society for Microbiology (CA-SFM^1^, last consultation: 10 November 2018). We used *E. coli* reference strain ATCC 25922 as a control. Isolates with a negative synergy test were considered as producer of plasmid-mediated cephalosporinases. Hence, isolates that grow on ESBL selective plates (i.e., resistant to 3GC) were sorted into ESBL or plasmid-mediated cephalosporinase producers. For all ESBL-producing isolates, we identified *bla*_ESBL_ genes by PCR and sequencing. We first screened the isolates carrying *bla*_CTX–M_ with consensus primers and we then targeted the different groups of CTX-M with specific primers (*bla*_CTX–M–group 1_, *bla*_CTX–M__–__group 9_, *bla*_CTX–M__–__group 2_). *bla*_CTX–M_ negative isolates were then tested for the presence of *bla*_SHV_ and *bla*_TEM_ genes (Mabilat and Courvalin, 1990; Cao et al., 2002; Coque et al., 2008). The nucleotide sequences of all PCR products were determined.

### Molecular Typing

Bacterial DNA was extracted from an overnight culture on Mueller-Hinton agar (Bio-Rad, Marne la Coquette, France) using the boiling method. We used the Clermont method to assign all the isolates to one of the eight existing phylogroups (A, B1, B2, C, D, E, F, G) (Clermont et al., 2013, 2019). Phylogroups were assigned depending on the presence/absence of four genes (*arpA*, *chuA*, *yjaA*, and TspE4.C2) using a quadruplex PCR. Isolates with identical profiles were further discriminated by additional PCRs. Hence, we amplified *trpA* to differentiate the isolates in phylogroups A and C, *arpA* to differentiate the isolates in phylogroups D and E, and *cfaB* and *ybgD* to differentiate isolates of the phylogroups B2, F, and G. Particular profiles allowed us to differentiate *E. coli* and *Escherichia* cryptic clades (Clermont et al., 2013). Isolates belonging to phylogroups B2 or D were typed by multi-locus sequence typing (MLST) using a previously described method (Wirth et al., 2006).

### Sequence Types Distribution

The distribution of STs of interest was assessed with an SNP-based analysis of the concatenation of the seven alleles of 4195 STs extracted from EnteroBase (Alikhan et al., 2018). We built a phylogenetic tree with FastTree v2.1.9 (Price et al., 2009, 2010), with a GTR-G model to visualize the links between the different STs. Subtrees were then extracted using the R package ggtree (Yu et al., 2017, 2018) to precisely visualize the population structure of phylogroups B2 and D. STs found in clinical settings or in treated wastewater were extracted from previously published results (Gibreel et al., 2012; Bréchet et al., 2014; Kallonen et al., 2017). Their distribution on the trees was compared with that of the isolates retrieved from the floodplains.

### Statistical Analysis

The independence of the explanatory variables of the whole dataset (i.e., spatial – floodplain and site variables) was tested using Pearson’s χ^2^-tests on the contingency tables of each pair of explanatory variables. The explanatory variables were all independent of each other. Therefore, the independence of (*i*) the phylogroups and (*ii*) the relationships between phylogroups mostly associated to human (B2, D, F) and other (A, B1, C, E) *E. coli* strains with respect to each explanatory variable (one by one) was tested. We first separately constructed contingency tables for (*i*) the phylogroups and each explanatory variable and (*ii*) the association or not of the isolates with humans and each explanatory variable for *E. coli* cultured on DRIGs or ESBL selective plates. We then performed Pearson’s χ^2^-tests on each contingency tables. The α value was set to 0.05. All analyses were performed with R 3.5.1 software (R Core Team Package 2019).

## Results

We retrieved 179 isolates of *E. coli*. We cultured 125 isolates on DRIGs; these isolates represented the unselected *E. coli* population present in the different rivers and their associated wetlands. We also cultured water samples on selective plates and identified 54 3GC-R isolates of which 49 and 5 produced an ESBL and a plasmid-mediated cephalosporinase, respectively. We also found 29 isolates belonging to the *Escherichia* cryptic clades (Clermont et al., 2013).

### Phylogroup Distribution of the Unselected *E. coli* Population

**FIGURE 1:**
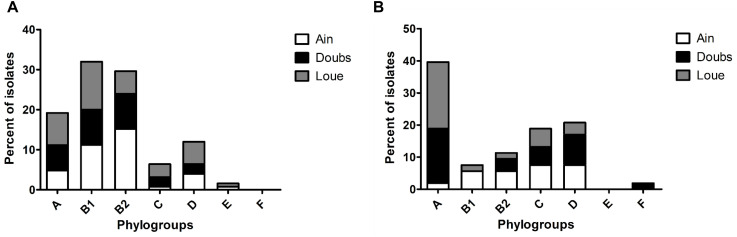
Phylogroup distribution among the isolates retrieved from the three floodplains in eastern France. **(A)** Phylogroup distribution of the overall population of *E. coli* according to the floodplain. **(B)** Phylogroup distribution of 3GC-R *E. coli* according to the floodplain.

The phylogroup distribution of the unselected *E. coli* population (cultured on DRIGs) showed that isolates belonging to phylogroups B1 (32%) and B2 (29.6%) were predominant (Figure 1A), with a high proportion of B2 isolates in the Ain floodplain (*p* = 0.048). Isolates belonging to phylogroups A (19.2%), C (6.4%), and D (12%) were identified in each floodplain, whereas isolates from phylogroup E (0.8%) were identified from only the Ain floodplain.

### 3GC-R *E. coli*

Compared to the other phylogroups, phylogroup A (39.6%) was dominant in 3GC-R *E. coli* (Figure 1B; *p* = 0.002). Phylogroups C (18.9%) and D (20.7%) were more frequent than phylogroups B2 (11.3%) and B1 (7.6%). Phylogroup B1 was found only in the Ain and Loue floodplains, whereas phylogroup F (1.8%) was only found in the Doubs floodplain. There were more isolates belonging to phylogroup A in the Loue and Doubs floodplains than in the Ain floodplain (*p* = 0.018). Isolates belonging to the phylogroups mostly associated to human (B2, D, and F) were less frequent than the other phylogroups (*p* = 0.019).

*bla*_ESBL_ in 54 3GC-R isolates were mostly *bla*_CTX–M__–__15_ (43%) and *bla*_CTX–M__–__1_ (24%). *bla*_CTX–M__–__14_ and *bla*_CTX–M__–__27_ represented 17 and 7% of the *bla*_ESBL_ genes, respectively. The remaining 3-GC-R isolates (9%) presumably produced a plasmid-mediated cephalosporinase. Among the isolates belonging to the phylogroups mostly associated to human (B2, D; *n* = 17), 47, 29, 12, and 12% produced CTX-M-15, CTX-M-14, CTX-M-1, and plasmid-mediated cephalosporinase, respectively.

### MLST Typing of *E. coli* Belonging to Phylogroups B2 and D

All phylogroup B2 and D isolates (*n* = 69) were typed by MLST. We obtained 31 different STs, nine of which were new STs (Supplementary Table 1). Twenty-six isolates belonged to STs often associated with humans (ST10, ST12, ST38, ST69, ST73, ST95, ST131, ST372, ST405).

A phylogenetic tree comprising 4195 STs extracted from EnteroBase (Alikhan et al., 2018) was built. Then, we extracted the phylogroup B2 and phylogroup D subtrees (Figures 3A,B, respectively). The subtrees showed that the isolates from clinical or environmental collections (WWTPs or floodplains) were scattered throughout the trees, indicating (i) that the structure of these populations was comparable and (ii) the absence of a specific population in the environment.

Three phylogroup B2 STs (ST95, ST131, and ST372) and four phylogroup D STs (ST38, ST69, ST362, ST405) were shared among the three different collection groups.

### *Escherichia* Cryptic Clades

We retrieved 29 isolates belonging to *Escherichia* cryptic clades, which represents 14% of the isolates retrieved in this study. The *Escherichia* cryptic clades were predominant in wetlands not connected to rivers (*p* = 0.026).

## Discussion

### Phylogroup Distribution of the Unselected *E. coli* Population

We previously determined that the concentration of total *E. coli* was higher in the rivers than in the wetlands with no significant differences in concentrations between wetlands (Henriot et al., 2019). We then divided 125 *E. coli* isolates into phylogroups and found a high proportion of isolates belonging to phylogroups A and B2, representing half of the isolates retrieved (48.8%). It was unexpected to find mostly human-associated phylogroups in low-anthropized wetlands (Escobar-Páramo et al., 2004; Massot et al., 2016). Previous studies identified WWTPs, sewage sludge and manure as sources of *E. coli* contamination in the environment (Michael et al., 2013; Balière et al., 2015). The contamination of the wetlands by isolates belonging to phylogroups A and B2 probably resulted from the release of WWTP outflows and the spread of sewage sludge. Phylogroup B1 is the main phylogroup found in animals (Gordon and Cowling, 2003). The high proportion of isolates belonging to this phylogroup (32%) retrieved in the floodplains can be explained by direct contamination of the water by animals (wild animals or cattle, via superficial or groundwater flow) or by the spread of manure. Interestingly, the Ain floodplain was contaminated by a higher proportion of isolates belonging to phylogroup B2 than the Doubs and Loue floodplains (Figure 1A). This difference could be attributed to two concurrent factors in the Ain lower floodplain: a very coarse substrate (cobblestones) overlaid with a shallow soil layer and the spread of large amounts of sewage sludge. One can speculate that the spread of sewage sludge is a major source of contamination of the Ain River and wetlands by phylogroup B2 isolates, which are of human origin. However, this hypothesis needs to be confirmed by precise follow-up regarding the spread of sewage sludge and manure in each floodplain near the studied wetlands.

### Resistance and Phylogroup Distribution of 3GC-R *E. coli*

**FIGURE 2:**
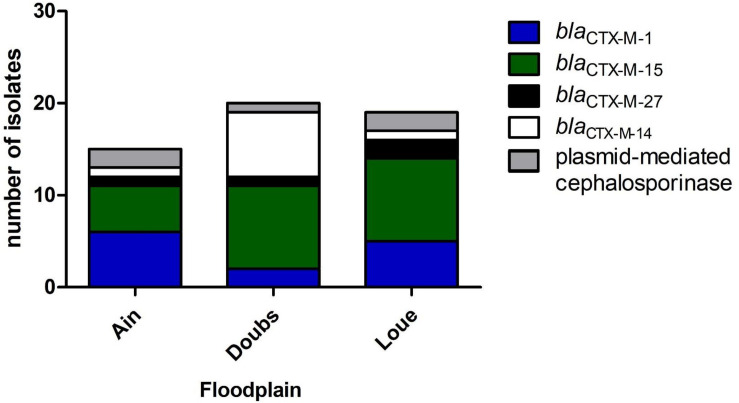
Distribution of the genes conferring resistance to 3-GC among the 3GC-R isolates retrieved from the three floodplains in eastern France (*n* = 54).

Phylogroup A was the main phylogroup found in 3GC-R *E. coli* (39.6%). These data are in line with the proportion of phylogroup A isolates recovered from humans (Tenaillon et al., 2010). This high proportion of isolates belonging to phylogroups A (39.6%) and B2 (11.3%) and the lower proportion of phylogroup B1 isolates (7.6%) suggest that the contamination of rivers and wetlands by 3GC-R *E. coli* could be caused more by human contamination rather than cattle contamination. The identification of *bla*_ESBL_ genes showed that *bla*_CTX–M__–__15_ was predominant, accounting for 43% of the *bla*_ESBL_ identified in the 3GC-R isolates (Figure 2). *bla*_CTX–M__–__27_ was present in 7% of the isolates. Together, *bla*_CTX–M__–__15_ and *bla*_CTX–M__–__27_ represented half of the *bla*_ESBL_ found. These *bla*_ESBL_ are highly related to *E. coli* found in humans (Day et al., 2019; Ludden et al., 2019). *bla*_CTX–M__–__14_ has previously been documented as associated to humans (Mughini-Gras et al., 2019), but this ESBL-encoding gene was also found in animals, especially in chicken (Hayashi et al., 2018). In contrast, *bla*_CTX–M__–__1_ is more often related to animals and meat (Day et al., 2019). This indicated that 3GC-R *E. coli* that contaminate the floodplains mostly originated from human effluents released from WWTP outflows into the rivers (Oberlé et al., 2012; Bréchet et al., 2014). Many studies identified multidrug-resistant *E. coli* in rivers downstream large cities (Dhanji et al., 2011; Jang et al., 2013). Here, we found multidrug-resistant *E. coli* in areas that were far from any direct contamination by humans (i.e., up to 20 km downstream from the WWTP outflows in our case), suggesting that these isolates can survive in rivers despite long-distance dispersal by water.

### *Escherichia* Cryptic Clades

The cryptic clades of *E. coli* comprise five different groups. Clade I is considered a divergent *E. coli* and has been reported in human infections, while the other clades are less virulent than clade I (Clermont et al., 2013). These cryptic clades are phenotypically indistinguishable from *E. coli* but genetically different.

The 29 isolates belonging to the cryptic clades retrieved in our study represented 14% of the isolates found. Previous studies found similar proportions of these isolates in environmental samples in Australia, France, and Italy (Walk, 2015). We found that these clades were frequent in unconnected wetlands which are supplied by groundwater, nutrient-poor, and cold (Bornette and Large, 1995; Bornette et al., 1996). In fact, low chlorophyll-a content, which directly relates to lower phytoplankton abundance and nutrient availability (Arthaud et al., 2013), and low temperature were nearly significantly associated with the presence of cryptic clades in wetlands of our study (data not shown). These cryptic clades may be genetically adapted to the environment by the presence of genes promoting their survival in this ecological niche (Walk, 2015).

### MLST Typing

Among the B2 and D isolates, we found ST73, ST131, ST95, ST69, and ST12. These STs are predominant in isolates retrieved in the patient blood samples (Kallonen et al., 2017), further suggesting the human origin of the contamination.

**FIGURE 3:**
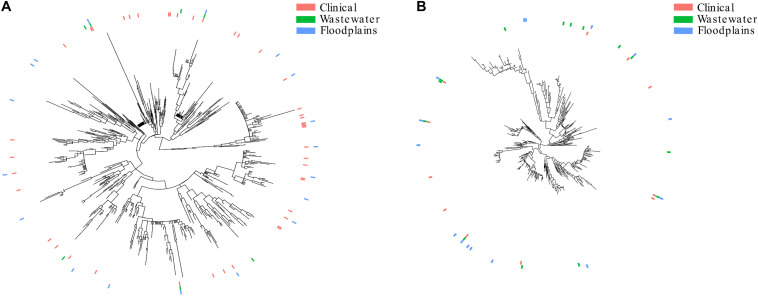
Population structure of *E. coli* retrieved from the floodplains, clinical settings and wastewater treatment plants. **(A)** Subtree of the isolates belonging to phylogroup B2. **(B)** Subtree of the isolates belonging to phylogroup D. The presence of STs in clinical settings, WWTPs and floodplains are represented by pink, green and blue lines, respectively.

The population structure of the present collection of *E. coli* was compared to that of collections from WWTP outflows and clinical settings (Figures 3A,B). Although not fully representative of all STs present in clinics or WWTPs, we found a comparable distribution of STs in the three collections that were distributed throughout the trees, suggesting that no specific sub-population of *E. coli* is adapted to a given environment. The similar distribution of STs in the WWTP outflows and in the environment strongly suggests that the contamination of the environment mostly results from the dilution of WWTP outflows. It also suggests that these isolates can reach and survive in remote areas like wetlands.

### *E. coli* Survival in Water

Our results showed that low-anthropized wetlands contain *E. coli* isolates of human origin. Although *E. coli* contamination of rivers or lakes has been well-documented (Michael et al., 2013), that of wetlands has been less investigated. Indeed, the studied wetlands have varying degrees of connection with the river, can be supplied in water either by the river or by groundwater, and the physical and chemical parameters can vary from those of the river (Henriot et al., 2019). It has been reported that *E. coli* can survive and grow in the environment (Jang et al., 2017). However, its survival is influenced by many factors, such as the carbon sources, temperature, pH, and availability of water (van Elsas et al., 2011). Here, we collected human-associated and 3GC-R isolates from a wide range of conditions (see Henriot et al., 2019 for details). Continuous discharge from WWTPs could explain the high proportion of human-associated *E. coli* isolates retrieved in low-anthropized environments. Previous studies suggested the existence of environmentally-adapted *E. coli* populations (van Elsas et al., 2011; Jang et al., 2017). Multi-drug resistant *E. coli* have been found in mangrove estuaries (Ghaderpour et al., 2015). The contamination of this low-anthropized environment was attributed to antibiotic release and human activities. Petit et al. (2017) found different distributions of *E. coli* population depending on the presence of cattle farm near the river. Overall, *E. coli* from human and animal effluents do not appear to be disadvantaged in environments and specific areas such as wetlands or mangrove estuaries which features drastically differ from those in the vertebrate intestine. In other words, the *E. coli* population in wetlands seems to be a dilution of the *E. coli* population found in human and animal guts. It reflects either the survival of *E. coli* of mammal origin in the environment and/or the permanent release of these contaminants in the environment. As the phylogrouping does not fully overlap the isolate origin, we cannot track the sources of the bacterial contamination with certainty.

## Conclusion

We found that a high proportion of the *E. coli* isolates retrieved in low-anthropized wetlands were probably human-associated. The similar population structures found in clinical settings, WWTPs and the environment suggest that *E. coli* released in the environment can reach and survive in specific areas like wetlands.

## Data Availability Statement

All datasets presented in this study are included in the article/Supplementary Material.

## Author Contributions

DM, CH, GB, XB, and DH conceived and designed the experiments and wrote the manuscript. DM, MB, JC, and MR carried out the experiments. DM, CH, CC, and BV analyzed the data. All authors contributed to the article and approved the submitted version.

## Conflict of Interest

The authors declare that the research was conducted in the absence of any commercial or financial relationships that could be construed as a potential conflict of interest.

## Footnotes

1 http://www.sfm-microbiologie.org/
