# Supplementary material for: High Prevalence of Human-Associated Escherichia coli in Wetlands Located in Eastern France
Source: Front Microbiol. 2020 Sep 4;11:552566. doi: 10.3389/fmicb.2020.552566 (PMC7498643; doi:10.3389/fmicb.2020.552566)
Supplement: Supplementary file 1 [file Data_Sheet_1.docx]

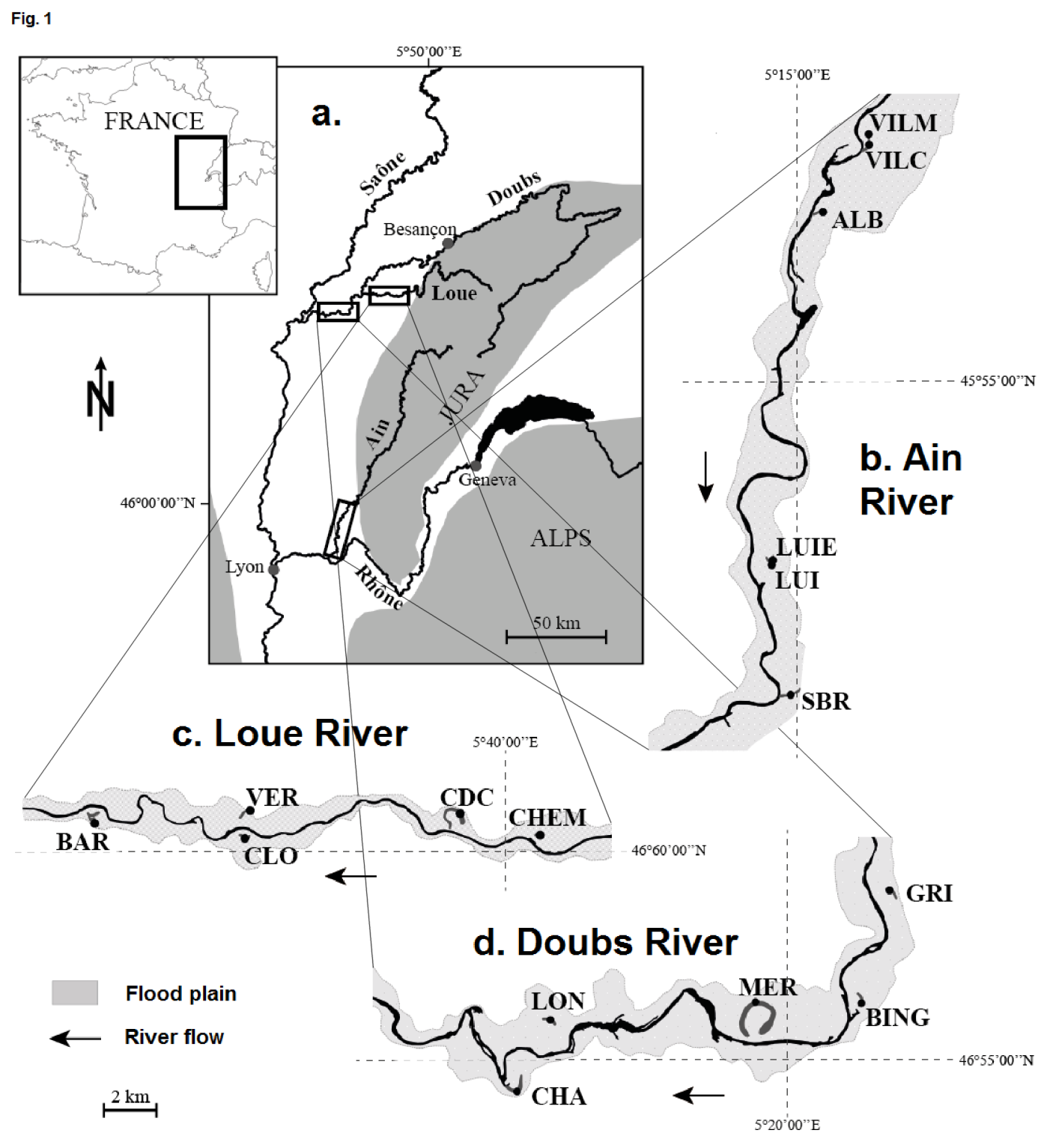


**Supplementary Figure S1.** Location of the 16 studied wetlands. The top left panel represents the locations of the three considered hydrosystems in France. (A) Location of the river network under study on the map of France. (B) Detail of the river Ain floodplain. (C) Detail of the river Ain floodplain. (D) Detail of the river Doubs flood plains.
